# Lupeol Attenuates Oxysterol-Induced Dendritic Cell Activation Through NRF2-Mediated Antioxidant and Anti-Inflammatory Effects

**DOI:** 10.3390/ijms26157179

**Published:** 2025-07-25

**Authors:** Sarmistha Saha, Antonella Capozzi, Elisabetta Profumo, Cristiano Alessandri, Maurizio Sorice, Luciano Saso, Brigitta Buttari

**Affiliations:** 1 Department of Cardiovascular, Endocrine-Metabolic Diseases and Aging, Italian National Institute of Health, 00161 Rome, Italy; sarmistha_pharmacol@yahoo.com (S.S.); elisabetta.profumo@iss.it (E.P.); 2 Department of Biotechnology, Institute of Applied Sciences & Humanities, GLA University, Mathura 281406, India; 3 Department of Experimental Medicine, Sapienza University of Rome, 00185 Rome, Italy; antonella.capozzi@uniroma1.it (A.C.); maurizio.sorice@uniroma1.it (M.S.); 4 Rheumatology Unit, Department of Clinical Internal, Anesthesiological and Cardiovascular Sciences, Sapienza University of Rome, 00185 Rome, Italy; cristiano.alessandri@uniroma1.it; 5 Department of Physiology and Pharmacology Vittorio Erspamer, Sapienza University of Rome, 00185 Rome, Italy; luciano.saso@uniroma1.it

**Keywords:** natural compound, dendritic cells, autoimmune diseases

## Abstract

Oxysterols such as 7-ketocholesterol (7KCh) contribute to the pathogenesis of autoimmune and chronic inflammatory diseases by inducing oxidative stress and promoting pro-inflammatory immune cell activation. Dendritic cells (DCs) play a central role in maintaining immune tolerance, and their dysregulation is a key driver of autoimmunity. Targeting DCs by using natural compounds offers a promising strategy to restore redox balance and suppress aberrant immune responses. This study investigated the immunomodulatory and antioxidant properties of Lupeol, a natural triterpenoid, in human monocyte-derived DCs exposed to 7KCh. Flow cytometry and cytokine profiling demonstrated that Lupeol preserved the immature, tolerogenic phenotype of DCs by promoting a dose-dependent increase in the anti-inflammatory cytokine IL-10. Lupeol also inhibited the 7KCh-induced upregulation of maturation markers (CD83, CD86) and suppressed the release of pro-inflammatory cytokines IL-1β and IL-12p70. Functionally, Lupeol-treated DCs directed T cell polarization toward an anti-inflammatory and regulatory profile while dampening the inflammatory responses triggered by 7KCh. This immunoregulatory effect was further supported by the decreased secretion of the pro-inflammatory cytokines IL-1β and IL-12p70 in DC culture supernatants. Mechanistic analyses using immunofluorescence showed that Lupeol alone significantly increased nuclear NRF2 levels and upregulated HO-1 expression. Western blot analysis further confirmed Lupeol’s ability to activate the KEAP1-NRF2 signaling pathway, as evidenced by increased expression of NRF2 and its downstream target, NQO1. The use of ML385, a selective NRF2 inhibitor, in ROS and cytokine assays supported the involvement of NRF2 in mediating the Lupeol antioxidant and anti-inflammatory effects in DCs. Notably, the oxidative burden induced by 7KCh limited the full activation of NRF2 signaling triggered by Lupeol. Furthermore, docking and MM/PBSA analyses revealed the specific interactions of Lupeol with the kelch domain of KEAP1. These findings suggest that Lupeol may serve as a promising orally available immunomodulatory agent capable of promoting tolerogenic DCs, offering potential applications in autoimmune and other chronic inflammatory diseases.

## 1. Introduction

Autoimmune diseases constitute a clinically heterogeneous group of over 80 disorders characterized by a breakdown in immunological self-tolerance, leading the immune system to mount aberrant responses against self-antigens [1]. These disorders, which typically manifest in early adulthood and are more common in women, significantly impair quality of life and are associated with elevated morbidity and mortality. The chronic and relapsing nature of autoimmune disorders also imposes a considerable burden on healthcare systems.

Emerging evidence implicates oxysterols—oxidized cholesterol derivatives—as key modulators of immune responses and contributors to the pathogenesis of several auto-immune conditions, particularly those involving the central nervous system, inflammatory bowel disease, and rheumatoid arthritis [2]. These immunomodulatory effects are mediated through both liver X receptor-dependent and independent pathways. Among oxysterols, 7-ketocholesterol (7KCh) has shown a profound influence on cytokine expression and monocytic cell differentiation [3,4], positioning oxysterol signaling as a promising therapeutic target in autoimmunity.

While pharmacological therapies remain central to autoimmune disease management, non-pharmacological strategies—particularly nutritional interventions—are gaining attraction for their ability to limit chronic inflammation and oxidative stress, potentially reducing long-term healthcare costs [5].

Natural compounds, especially phytochemicals, have emerged as promising adjunct therapies due to their capacity to improve endothelial function, reduce arterial stiffness, and exert potent anti-inflammatory and antioxidant effects [6,7], including in autoimmune settings. Their safety and tolerability are supported by epidemiological and clinical studies [8,9], although further research is required to validate efficacy and elucidate molecular mechanisms.

Plant-derived bioactives, particularly polyphenols and flavonoids abundant in Mediterranean diets, have demonstrated strong immunomodulatory potential [10,11,12,13,14]. These compounds modulate immune responses, reduce disease activity, and improve quality of life in individuals with autoimmune diseases [5,15].

Dendritic cells (DCs), the most potent antigen-presenting cells of the innate immune system, are pivotal in shaping adaptive immune responses. By sensing environmental cues and presenting antigens to T cells, DCs determine the balance between immunity and tolerance. Various natural compounds have been shown to influence DC function, altering their maturation status, cytokine profiles, and antigen presentation capabilities. These effects provide a targeted approach for immune modulation, with potential applications in autoimmune diseases where restoration of immune tolerance is a key therapeutic challenge. Such strategies may offer safer alternatives to conventional immunosuppressive therapies by promoting immune homeostasis rather than generalized immune suppression [16,17,18].

Under homeostatic conditions, DCs can adopt a tolerogenic phenotype (TolDCs), which supports peripheral tolerance by deleting autoreactive T cells and promoting regulatory T cell responses, especially through modulation of the Th17/Treg balance [19,20]. TolDCs are characterized by reduced co-stimulatory molecule expression [21], high IL-10 production [22], T cell-polarizing activity toward a regulatory phenotype [23], and a distinct immunometabolic profile that supports their suppressive function [24]. In contrast, inflammatory environments drive DCs toward a pro-inflammatory state, perpetuating immune activation and contributing to autoimmune disease progression [25].

Recent studies have shown that certain plant-derived compounds can promote TolDC-like features, helping restore immune homeostasis and reduce inflammation in autoimmune settings [26]. These bioactive phytochemicals influence the differentiation and function of both conventional and plasmacytoid DCs, favoring their tolerogenic reprogramming [27,28,29,30].

In inflammatory conditions, DCs pretreated with polyphenols exhibit attenuated pro-inflammatory signaling and enhanced activation of cytoprotective pathways. These effects are largely mediated by NRF2 (nuclear factor erythroid 2-related factor 2), a transcription factor that regulates antioxidant responses and modulates inflammation [28,31]. Some phytochemicals also activate AMP-activated protein kinase, which inhibits mTOR signaling in LPS-stimulated DCs, further enhancing immunoregulatory functions [27].

Among these natural compounds, Lupeol, a triterpenoid present in various fruits, vegetables, and medicinal plants, has gained attention for its broad-spectrum antioxidant and anti-inflammatory activities, primarily mediated via NRF2 activation and induction of the cytoprotective enzyme heme oxygenase-1 (HO-1) [32,33]. Given NRF2’s central role in maintaining redox homeostasis and regulating immune responses, targeting this pathway offers a promising strategy for immune intervention [34]. While NRF2 activation and DC modulation are broadly relevant to various inflammatory conditions, the present study emphasizes their prospective relevance for autoimmune disease contexts, particularly considering the well-established contribution of oxysterols like 7KCh in the pathogenesis of several autoimmune disorders [2,5].

Despite evidence of Lupeol’s anti-inflammatory activity in macrophages, its effects on human DCs and NRF2 signaling remain unexplored. Prompted by this gap in knowledge and Lupeol’s potential to activate the NRF2 pathway, the present study aimed to investigate whether Lupeol promotes the differentiation of TolDCs. Using immunochemical and flow cytometric analyses, we investigated the ability of Lupeol to reprogram human monocyte-derived DCs toward a tolerogenic phenotype, focusing on changes in surface markers, cytokine expression, and underlying molecular mechanisms.

## 2. Results

### 2.1. Lupeol Induces Tolerogenic DCs and Counteracts the 7KCh-Inflammatory Activity

**Figure 1:**
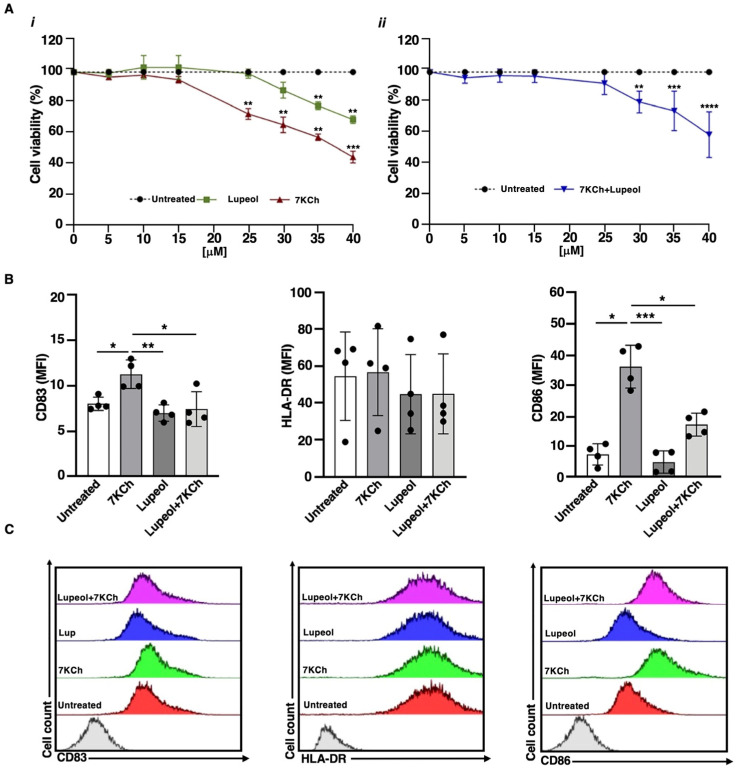
(**A**) Viability of immature monocyte-derived dendritic cells (iDCs) after in vitro exposure to Lupeol and 7-ketocholesterol (7KCh). The percentage of viable cells was assessed by trypan blue exclusion after 18 h of exposure to (*i*) Lupeol or 7KCh (0–40 μM) or (*ii*) their combination [7KCh (15 μM) + Lupeol (0–40 μM)]. Data represent the mean ± SD of three independent experiments. Dotted lines represent untreated cells (100% viable cells). Statistical significance was determined by unpaired *t*-test: ** *p* < 0.01; *** *p* < 0.001; **** *p* < 0.0001 vs. control. (**B**) Surface marker expression on immature monocyte-derived dendritic cells (iDCs) after in vitro exposure to Lupeol and 7KCh. Immature DCs (8 × 10^5^ cells/mL) were stimulated with or without Lupeol (25 µM) and 7KCh (15 µM) or both for 18 h, and then a four-color flow cytometry analysis was performed to evaluate surface marker expression. Histograms show the mean fluorescence intensity (MFI) of CD83, HLA-DR, and CD86 expression. (**C**) Representative two-dimensional flow cytometry plot images showing the fluorescence intensity of surface markers under different conditions. Results are expressed as mean value ± SD of four independent experiments. Significance was determined by one-way ANOVA followed by Tukey’s post hoc analysis; * *p* < 0.05; ** *p* < 0.01; *** *p* < 0.001.

To evaluate the impact of Lupeol and 7KCh on immature monocyte-derived DC (iDC) characteristics, we first assessed their cytotoxicity using the trypan blue exclusion assay (Figure 1A). iDCs were exposed for 18 h to increasing concentrations of Lupeol (0–40 µM), 7KCh (0–40 µM), or their combination (7KCh 15 µM + Lupeol 0–40 µM). As shown in Figure 1A(*i*), Lupeol alone induced a slight concentration-dependent reduction in cell viability, with a ~20% decrease at 35 µM and ~35% decrease at 40 µM.

In contrast, 7KCh led to a ~25% reduction in viability at 25 µM, which further declined to ~50% at 40 µM. When combined with 7KCh (15 µM), Lupeol did not change cell viability at the 5–25 µM dose range, while it significantly reduced the percentage of viable cells at the 30–40 µM dose range (Figure 1A(*ii*)), identifying 25 µM as the optimal concentration for subsequent experiments. We analyzed the expression of key maturation markers using flow cytometry after 18 h of treatment. Specifically, we measured CD83, a marker of DC maturation, along with the costimulatory molecules CD86 and HLA-DR. Similar to untreated iDCs, those exposed to Lupeol displayed an immature phenotype, characterized by low levels of CD83, HLA-DR, and CD86, as indicated by mean fluorescence intensity (MFI; see Figure 1). Stimulation of iDCs with 7KCh promoted DC maturation, as evidenced by elevated CD83 and CD86 levels (see Figure 1) relative to untreated iDCs. However, iDCs co-treated with Lupeol and 7KCh exhibited a lower degree of activation than those treated with 7KCh alone (see Figure 1).

### 2.2. Lupeol-Treated iDCs Prime Naïve T-Cells into Anti-Inflammatory and Regulatory Cells

**Figure 2:**
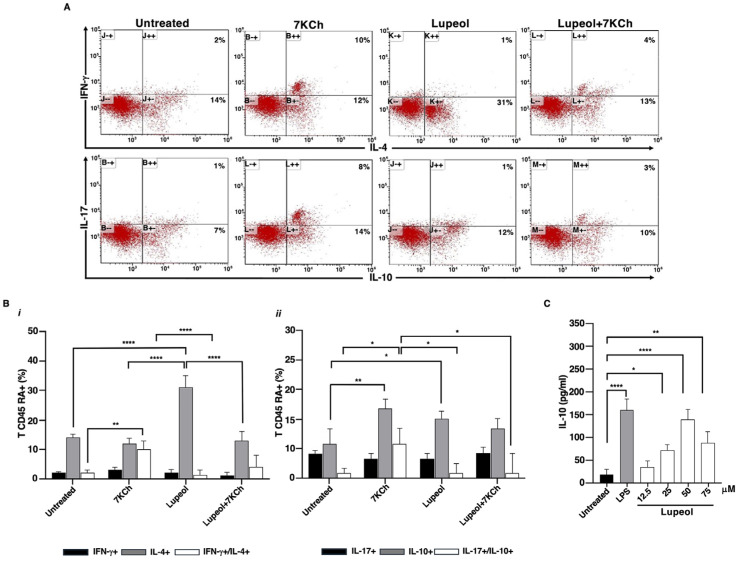
Lupeol-treated DCs stimulated allogeneic naive human T cells to produce IL-4 and IL-10. (**A**). Five-day human DCs were stimulated with or without Lupeol (25 µM) and 7KCh (15 µM) for 18 h. Negatively selected naïve allogeneic T cells were co-cultured with the treated DCs at a 20:1 ratio for 10 days and then the cells were stained with anti-hu-CD3PerCP and processed for intracellular labeling with anti-hu-IFN-γ-APC and anti-hu-IL-4-PE or anti-hu-IL-17-APC anti-hu-IL-10-PE. The numbers into the dotplots show the percentage of activated CD3+ cells producing the cytokine. The figure shows a representative experiment from 3 with similar results. (**B**) Histograms show the percentage of activated allogeneic CD3+ CD45RA+ T cells producing the IFN-γ and IL-4 cytokines (*i*) and the IL-17 and IL-10 cytokines (*ii*) analyzed by flow cytometry. (**C**) Dose-response production of IL-10 in Lupeol-stimulated DC culture supernatants after 18 h was analyzed by specific ELISA experiments. Results are expressed as mean value ± SD of 3 independent experiments. Significance was determined by one-way ANOVA followed by Tukey’s post hoc analysis; * *p* < 0.05; ** *p* < 0.01; **** *p* < 0.0001.

In cell culture experiments designed to find out whether Lupeol-treated iDCs primed and polarized allogeneic naïve CD4+ CD45RA+ into a pro-inflammatory or regulatory phenotype, cytofluorimetric analysis showed that most naïve T cells cocultured with Lupeol-treated iDCs turned into IL-4 and IL-10 producing T cells (see Figure 2A,B).

Conversely, 7KCh-treated iDCs showed a larger number of IFN-γ+/IL-4+ and IL-17+/IL-10+ T cells compared to unstimulated iDCs (see Figure 2A,B). Notably, DCs exposed to both stimuli exhibited a few IL-17+/IL-10+ T cells.

The bias towards an anti-inflammatory and regulatory phenotype after stimulation with Lupeol was supported by dose-dependent IL-10 production that resulted in higher culture supernatants from Lupeol-treated iDCs than in those from untreated iDCs (Figure 2C).

### 2.3. Lupeol Modulates Oxidative Stress and Cytokine Production in Dendritic Cells via NRF2 Activation

**Figure 3:**
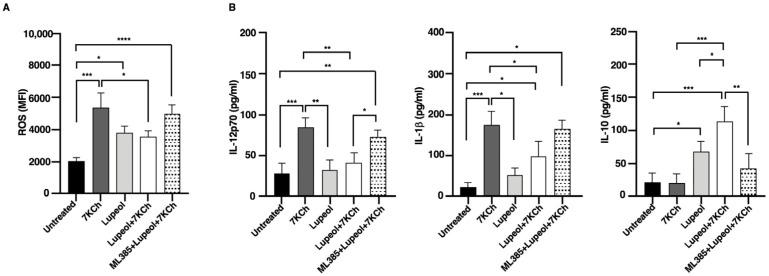
Flow cytometry analysis of intracellular ROS and cytokine production in immature monocyte-derived dendritic cells (iDCs). (**A**) The histogram shows media and standard deviations of the mean fluorescence intensity (MFI) of ROS production after 18 h analyzed by flow cytometry in treated or untreated iDC. (**B**) Cytokine production in supernatants collected after 18 h was analyzed by specific ELISA experiments in treated or untreated iDC. Significance was determined by one-way ANOVA followed by Tukey’s post hoc analysis; * *p* < 0.05; ** *p* < 0.01; *** *p* < 0.001; **** *p* < 0.0001.

To assess intracellular ROS production, both untreated and treated iDCs were stained with H_2_DCF-DA and analyzed by flow cytometry. The results showed a significantly higher MFI in iDCs treated with either 7KCh or Lupeol compared to control iDCs. However, ROS production was notably lower in Lupeol-treated iDCs than in those treated with 7KCh alone (Figure 3A).

Co-treatment with Lupeol and 7KCh resulted in a significant reduction in ROS levels compared to 7KCh-treated iDCs, suggesting that Lupeol exerts a partial protective effect against oxidative stress. To further investigate the molecular mechanism underlying this effect, we used ML385, a selective inhibitor of NRF2, which impairs NRF2’s ability to regulate oxidative stress responses. Notably, ML385 treatment abolished the antioxidant effect of Lupeol, as ROS levels in the combined group (ML385+Lup+7KCh) were significantly increased compared to untreated control DCs. However, ROS levels in this combined group did not differ significantly from those in DCs treated with Lupeol and 7KCh alone. To gain further insights into the immunomodulatory effects of Lupeol on DC activation, we examined cytokine production in DC supernatants. After 18 h of culture, DCs stimulated with 7KCh showed increased secretion of the pro-inflammatory cytokines IL-12p70 and IL-1β (Figure 3B). Notably, treatment with Lupeol attenuated this inflammatory response by downregulating IL-12p70 and IL-1β production while upregulating IL-10 secretion (Figure 3B), thereby promoting a shift toward an immunoregulatory DC phenotype. To determine whether the Lupeol-mediated cytokine modulation was NRF2-dependent, we again employed ML385. The reversal of Lupeol effects upon ML385 treatment, marked by a significant increase in IL-12p70 and a significant decrease in IL-10 levels when comparing the combined group (ML385+Lup+7KCh) to the Lupeol+ 7KCh group suggests that NRF2 activation is crucial for Lupeol’s regulatory role in DC function, although IL-1β levels were not significantly affected. These findings strengthen the hypothesis that Lupeol exerts its anti-inflammatory and immunomodulatory effects through NRF2 activation, modulating both oxidative stress and cytokine production in DCs. However, the oxidative and inflammatory burden imposed by 7KCh may limit Lupeol’s ability to sustain NRF2 activation under this condition.

### 2.4. NRF2 Activation Underlies Lupeol Modulation of DC Maturation

**Figure 4:**
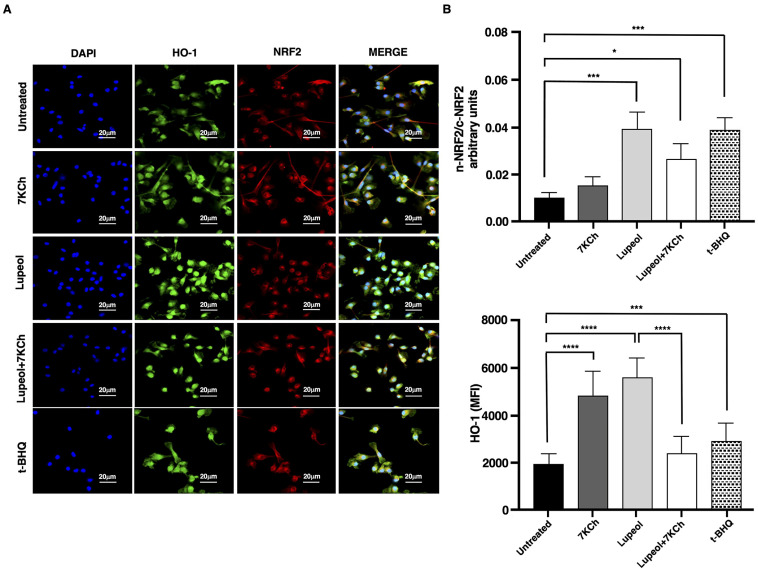
Analysis of NRF-2 pathway in immature monocyte-derived dendritic cells (iDCs) treated with Lupeol. (**A**) Representative immunofluorescent staining of NRF2 and HO-1 in iDCs treated with Lupeol (25 µM), 7KCh (15 µM) or tertbutylhydroquinone (t-BHQ) for 18 h. The upright microscope Nikon Eclipse Ni-U with 60× magnification was used to capture micrographs. (**B**) Quantification of the intensity of the fluorescence signal for HO-1 and NRF2 by using Image J 1.53 t software. Nuclei were counterstained with DAPI, and nuclear segmentation was performed for each field of view using the DAPI fluorescence signal. Whole-cell fluorescence intensities of HO-1 and NRF2 were quantified by analyzing their respective signals in the green and red channels. To assess NRF2 translocation dynamics, nuclear and cytosolic NRF2 signals were delineated based on their corresponding fluorescence in the previously segmented compartments. The translocation of NRF2 was determined by calculating the ratio of mean pixel intensity between the nuclear (n) and cytoplasmic (c) compartments (n-NRF2/c-NRF2 ratio). Data are presented as the mean ± SD for each group (*n* = 3). Significance was determined by one-way ANOVA followed by Tukey’s post hoc analysis; * *p* < 0.05; *** *p* < 0.001; **** *p* < 0.0001.

In consideration of the pivotal role carried out by NRF2 in controlling the expression of antioxidant genes that ultimately exert anti-inflammatory functions [34], we investigated the activation level of this transcription factor in iDCs exposed to Lupeol to evaluate the role of NRF2 in Lupeol modulation of DC maturation. The immunofluorescence images showed that at 24 h Lupeol, similarly to tertbutylhydroquinone (t-BHQ), a positive inducer of NRF2, triggered NRF2 activation, which was fully expressed in nuclei (Figure 4A). This upregulation of NRF2 levels was associated with an increase in expression of the NRF2 target gene HO-1 at 18 h (Figure 4B). However, when Lupeol was administered in combination with 7KCh, the increase in NRF2 nuclear levels and HO-1 expression appeared less pronounced, suggesting that the oxidative and inflammatory environment induced by 7KCh may attenuate Lupeol’s ability to sustain NRF2 activation.

**Figure 5:**
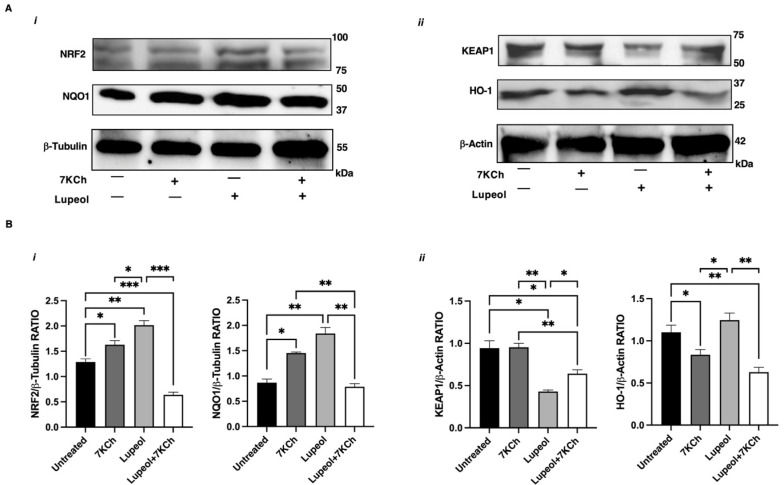
Analysis of NRF-2 pathway in immature monocyte-derived dendritic cells (iDCs) treated with Lupeol. (**A**) Representative Western blot results for NRF2 and NQO1 (*i*), and for KEAP1 and HO-1 (*ii*) in the whole-cell lysates of iDCs treated with 7KCh or Lupeol or both for 18 h. (**B**) Densitometric analysis of NRF2 and NQO1 panel (*i*) and KEAP1 and HO-1 panel (*ii*) in iDCs. Analyses were performed with National Institutes of Health ImageJ 1.62 software and normalized to the appropriate housekeeping proteins β-tubulin or β-actin. Normalized band intensities are expressed as the ratio of target protein to housekeeping control as mean ± SD of 3 independent experiments. Significance was determined by one-way ANOVA followed by Tukey’s post hoc analysis; * *p* < 0.05; ** *p* < 0.01; *** *p* < 0.001.

By using Western blot analysis, we evaluated the overall status of the NRF2 pathway and confirmed that Lupeol upregulated NRF2 expression (Figure 5A(*i*),B), and induced a decrease of KEAP1 levels (Figure 5A(*ii*)), leading to increased NQO1 protein expression (Figure 5A(*i*),B).

These findings confirm that the antioxidant and immunomodulatory effects of Lupeol are dependent on NRF2. However, when Lupeol was administered in combination with 7KCh, the upregulation of NRF2 and its target gene NQO1 was attenuated, despite a sustained reduction in KEAP1 levels (Figure 5A,B). These findings suggest that the oxidative and inflammatory environment induced by 7KCh may hinder Lupeol’s ability to maintain effective NRF2 activation.

### 2.5. Molecular Docking Analysis of Lupeol with Kelch Domain of Keap1 Protein

**Figure 6:**
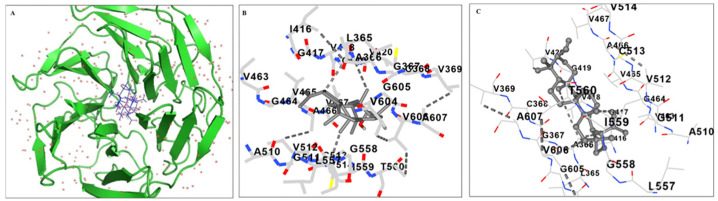
Molecular docking analysis. Docking and MM/PBSA analyses evaluating the specific interactions of lupeol with kelch domain of Keap1. (**A**) Ribbon representation of the KEAP1 protein (green) bound to the ligand Lupeol (gray sticks); surrounding red dots indicate water molecules, (**B**) Snapshot of the 3D- binding mode of the ligand with the protein and (**C**) Snapshot of the anchoring residues of kelch domain forming H-bonds with the ligand. Atoms are colored by element: oxygen (red), nitrogen (blue), and sulfur (yellow); dashed lines represent hydrogen bonds.

A ubiquitin ligase complex uses Keap1 as a substrate adaptor protein to bind the NRF2 transcription factor. According to these results, Lupeol enters the kelch domain of the KEAP1 cavity (Figure 6A), where it is stabilized by hydrophobic interactions and hydrogen bonding with KEAP1 residues.

Lupeol enters the cavity and engages in hydrophobic interactions with Val465, Ala466, Val604, and 510 (Figure 6B). Additionally, Lupeol makes hydrogen bonds with the backbone Ala366, Arg415, Ile416, Gly417, Val418, Val465, and Val512. Consequently, it was found that these interactions would help Lupeol anchor in the KEAP1 kelch domain. The calculated interactions and overall binding free energies obtained from the MM/PBSA calculations are listed in Table 1. The binding free energy for Lupeol in the kelch domain was found to be −56.01 ± 8.04 kcal/mol.

## 3. Discussion

This study provides novel insights into the immunomodulatory effects of Lupeol, a naturally occurring triterpenoid, demonstrating for the first time its direct impact on the phenotype and function of human DCs. At non-cytotoxic concentrations, Lupeol preserved the immature, tolerogenic phenotype of DCs, even in the presence of 7KCh, a highly pro-inflammatory oxysterol. These findings highlight Lupeol’s therapeutic potential in chronic inflammatory and autoimmune conditions.

Phenotypic analysis showed that Lupeol maintained low surface expression of key maturation markers (CD83, CD86, HLA-DR), consistent with a tolerogenic DC profile. In contrast, 7KCh promoted DC maturation, as evidenced by increased expression of CD83 and CD86, confirming its immunostimulatory role. These results are partially consistent with earlier work by Son et al. [35], who observed oxysterol-driven DC maturation in a different experimental setting. Discrepancies likely arise from differences in oxysterol concentration, treatment duration, and the use of primary cells versus immortalized lines. Nonetheless, our findings support the evidence that 7KCh promotes DC activation, reinforcing its pathogenic role in autoimmunity [2,36].

Functionally, although 7KCh did not significantly increase single-positive IFN-γ+ or IL-17+ T cells, it led to a higher frequency of IFN-γ+/IL-4+ and IL-17+/IL-10+ double-positive T cells. These double-positive populations may represent transitional or dysregulated T helper states, a phenomenon recognized in inflammatory and autoimmune contexts driven by T cell plasticity [37,38]. While a definitive Th1 or Th17 polarization was not observed, the marked increase in the secretion of pro-inflammatory cytokines IL-12p70 and IL-1β in the supernatants of 7KCh-treated DC cultures suggests a shift toward an inflammatory milieu. This is consistent with the well-documented immunostimulatory and pathogenic effects of oxysterols in autoimmune diseases [36,39]. In contrast, Lupeol-treated DCs promoted a shift toward an anti-inflammatory and regulatory profile in primed T cells, as indicated by an increase in single-positive IL-10+ and IL-4+ T cells and a reduction in IL-17+/IL-10+ double-positive T cells when DCs were co-treated with Lupeol and 7KCh. This immunoregulatory effect was further supported by the decreased secretion of the pro-inflammatory cytokines IL-1β and IL-12p70 in DC culture supernatants. These findings suggest that Lupeol-treated DCs favor the generation of an IL-10-enriched, anti-inflammatory environment. However, this likely reflects a general immunoregulatory shift rather than a definitive polarization toward classical Th2 or Treg lineages. Further studies will be required to precisely define the nature of T cell polarization, including the analysis of lineage-specific transcription factors and additional functional assays. Overall, these effects underscore Lupeol’s ability to inhibit DC-driven pro-inflammatory T cell activation and shift the immune response toward tolerance. Our findings are consistent with accumulating evidence indicating that certain bioactive food-derived compounds can induce tolerogenic reprogramming in DCs. For instance, Ano et al. [40] reported that the ergosterol analogue 14-dehydroergosterol significantly downregulated CD86 and HLA-DR expression in LPS-stimulated human monocyte-derived DCs, promoting tolerogenic features at a low concentration (<10 μM). Similarly, Vitamin D3 (VitD3) has been shown to modulate DC activation and promote anti-inflammatory responses [41], even though VitD3-induced TolDCs exhibited a slightly reduced viability and the exact mechanisms of how TolDCs are induced by vitD3 treatment have not been fully addressed until recently [42]. While these studies support the concept that natural compounds can exert immunomodulatory effects on DCs, differences in molecular structure, potency, concentration, and experimental design, including the type of inflammatory stimulus employed, likely account for the variability in DC modulation observed. This evidence highlights the heterogeneity of tolerogenic DC phenotypes and suggests that combinatorial approaches using different agents may further enhance the tolerogenic potential of DCs [43]. In this context, as an alternative approach and as supporting evidence, we also tried to find out the binding interactions of Lupeol on the kelch domain of the KEAP1 protein by docking studies. In an earlier study, Zhuang C. et al. [44] identified three important binding characteristics through their docking studies on noncovalent Keap1 inhibitors: salt bridge interactions, hydrogen bonds, and π-cation interactions. Furthermore, their research demonstrates how their aromatic scaffolds of inhibitors effectively integrate into the cationic Arg415 pocket. In our study, the aromatic ring of Lupeol is also lodged into Arg415, Gly417, and Val418. Moreover, the binding energy calculations also confirmed the interactions of Lupeol with the kelch domain. These results are in agreement with experimental results that showed the activation of the KEAP1-NRFf2 signaling pathway by Lupeol.

Extending previous observations of Lupeol’s anti-inflammatory and antioxidant activity in macrophages and other systems, our study demonstrates a similar effect in DCs. Mechanistically, Lupeol appears to modulate the KEAP1-NRF2 signaling axis, as shown by increased expression of canonical NRF2 targets such as HO-1 and NQO1. However, while Lupeol alone enhanced HO-1 and NQO1 expression, this effect was diminished in the presence of 7KCh, suggesting that the pro-oxidant and pro-inflammatory environment induced by 7KCh may interfere with sustained NRF2 activation. Our findings also indicate that Lupeol pre-treatment attenuates the secretion of pro-inflammatory cytokines (IL-12p70, IL-1β) and promotes IL-10 production in 7KCh-stimulated DCs, supporting a shift toward a tolerogenic phenotype. The use of ML385, a specific NRF2 inhibitor, partially reversed these effects; most notably, it significantly restored IL-12p70 levels and reduced IL-10 secretion. These results support a role for NRF2 in mediating the immunomodulatory effects of Lupeol. However, IL-1β levels were not significantly altered by ML385 treatment, and the reduction in ROS levels observed with Lupeol/7KCh co-treatment did not reach statistical significance when ML385 was added, highlighting the complexity of this regulatory axis. Taken together, these results suggest that while Lupeol exerts NRF2-dependent anti-inflammatory and immunomodulatory effects in DCs, the oxidative burden induced by 7KCh may limit the extent to which NRF2 signaling can be effectively activated under these conditions, potentially through feedback inhibition or crosstalk with competing inflammatory pathways [34]. Thus, the contribution of NRF2 to the observed effects warrants further investigation. The role of HO-1 in the tolerogenic reprogramming of DCs is well supported by previous studies showing that HO-1 induction contributes to the acquisition of anti-inflammatory and immunoregulatory phenotypes in DCs [45]. The observed increase in HO-1 expression following Lupeol treatment aligns with this immunoregulatory role and may contribute to the reduced release of IL-12p70 and IL-1β we detected in Lupeol-treated DCs. Our data on 7KCh-induced HO-1 expression are in line with the study by Jin et al. [46], which demonstrated that cholesterol treatment of vascular endothelial cells induced HO-1 expression via activation of the NRF2 and MAPK/ERK signaling pathways, likely reflecting a compensatory cytoprotective response to the oxidative stress generated by overlipid exposure. However, when combined with Lupeol, HO-1 expression appeared reduced compared to Lupeol alone, suggesting possible regulatory feedback mechanisms or differences in HO-1 induction pathways activated by 7KCh versus Lupeol.

Although direct evidence for NQO1 involvement in tolerogenic DC programming is limited, its induction by NRF2 likely contributes to the overall anti-inflammatory and redox-balanced environment that supports tolerogenic reprogramming, particularly in cooperation with other NRF2-regulated cytoprotective enzymes such as HO-1.

Further mechanistic studies are needed to dissect these regulatory dynamics, including experiments using HO-1 inhibitors or siRNA approaches to specifically evaluate HO-1’s contribution to DC tolerogenicity in this context. Additionally, future studies using NRF2 inhibitors in combination with HO-1 modulation would clarify the relative contributions of NRF2-dependent versus NRF2-independent mechanisms in driving DC reprogramming. Our findings using a pharmacological NRF2 inhibitor support a model in which Lupeol reprograms DCs toward a redox-balanced, anti-inflammatory phenotype by modulating both oxidative stress and immune activation pathways via NRF2 signaling. This aligns with previous studies on the immunoregulatory roles of triterpenoids and polyphenols through the NRF2/HO-1 axis [47,48,49]. However, further investigation of the role of the NRF2-ROS axis in DC-mediated T cell polarization is warranted. Importantly, the potential of Lupeol to induce tolerogenic DCs in situ through oral administration offers a practical alternative to ex vivo cell manipulation and reinfusion. This strategy could simplify clinical protocols, lower costs, and increase accessibility, while potentially extending drug-free remission periods in autoimmune diseases, as discussed by Schett et al. [50].

A limitation of this study is the use of monocyte-derived DCs, which, while widely accepted as an in vitro model, may not fully reflect the functional diversity and complexity of DCs in vivo. Future studies using tissue-resident or circulating DC subsets, as well as in vivo models, particularly in established autoimmune models, will be necessary to validate and extend these findings.

Future work should focus on validating whether the direct binding of Lupeol to KEAP1 may represent the main mechanism responsible for NRF2 activation via biophysical assays and on developing advanced delivery systems, such as Lupeol-loaded nanohydrogels, to enhance bioavailability and therapeutic efficacy in vivo.

## 4. Materials and Methods

### 4.1. In Vitro Culture of Human Monocyte-Derived Dendritic Cells, T Lymphocytes and Treatments

Peripheral blood mononuclear cells (PBMCs) were isolated from 6 buffy coats obtained from healthy blood donors (HDs) using density gradient centrifugation (Lympholite; Cedarlane, Hornby, ON, Canada). PBMCs were incubated with anti-CD14-coated microbeads (Miltenyi Biotec; Gladbach, Germany), and monocytes were sorted with the magnetic device MiniMacs Separation Unit (Miltenyi Biotec) according to the manufacturer’s instructions. Monocyte-derived DCs (termed immature DCs) were obtained by culturing adherent monocytes for 5 days in a complete medium [RPMI 1640 supplemented with 1% nonessential amino acids, 1% sodium pyruvate, 10,000 U/mL penicillin-streptomycin (Gibco, Karlsruhe, Germany), 5 × 10^−5^ M 2-mercaptoethanol (Merck, Darmstadt, Germany), and 10% fetal bovine serum (FBS, Hyclone Laboratories, Logan, UT, USA)] supplemented with 100 ng/mL recombinant human (rh) Granulocyte-Macrophage Colony-Stimulating Factor (GM-CSF) and 25 ng/mL rh interleukin-4 (IL-4). Trypan blue exclusion assay (Sigma-Aldrich, Milan, Italy) and light microscope (Nikon Eclipse Ni-U, Nikon Corporation, Tokyo, Japan) were used to assess cell viability and cell morphology, respectively. Immature DCs (DCs, 0.8 × 10^6^ cells/mL) were treated with or without Lupeol (1–50 µM; Sigma-Aldrich) for 1 h and then exposed to 7KCh (15 µM; Sigma-Aldrich) at 37 °C and 5% CO_2_ for a total of 18 h. Immature DCs were stimulated with 0.1 μg/mL lipopolysaccharide (LPS, from Escherichia coli strain 0111:B4, Sigma-Aldrich) to obtain control mature DCs in experiments evaluating IL-10 secretion. To exclude the possibility of endotoxin contamination in the reagents, experiments were also conducted in the presence of polymyxin B (10 μg/mL; Sigma-Aldrich). In some experiments, DCs were treated with ML385 (5 µM; S8790; Selleck, Houston, TX, USA), an NRF2 inhibitor. Untouched CD4+ naïve T cells were purified from PBMCs by magnetic selection using the Naïve CD4 T cell isolation kit II (Miltenyi Biotec), according to the manufacturer’s instructions. The purity of negatively selected naïve CD4+ T cells was more than 95%, as assessed by flow cytometric analysis.

### 4.2. Flow Cytometric Analysis of Phenotypic DC Maturation

Phenotypic surface markers were determined by staining DCs for 30 min at 4 °C with a panel of mouse anti-human monoclonal antibodies (mAbs): fluorescein isothiocyanate (FITC) anti-CD14, phycoerythrin (PE) anti-CD1a, Vioblue anti-CD86 (B7-2), PE anti-CD83, and Peridinin-Chlorophyll-Protein (PerCP)-Vio700 anti-human leukocyte antigen-D region-related (HLA-DR) (Miltenyi Biotec; Gladbach, Germany). Isotype-control antibodies served as negative controls. Cells were stained with Sytox^®^ Blue (Molecular Probes, Carlsbad, CA, USA) to allow the exclusion of dead cells during flow cytometric analysis. Data from 10,000 viable cells were acquired by a Gallios flow cytometer (Beckman Coulter, Brea, CA, USA), and data were analyzed with Kaluza Analysis Software v. 2.1 (Beckman Coulter).

### 4.3. Flow Cytometric Analysis of Reactive Oxygen Species (ROS) Production

The production of ROS in human DCs was measured through H2DCF-DA staining. In brief, DCs (1 × 10^6^ cells/mL) were incubated with H2DCF-DA at a final concentration of 2.5 μM. After 45 min of incubation in the dark at 37 °C, cells were centrifuged, and the pellet was washed twice with ice-cold phosphate buffered saline (PBS)/FBS and then fixed with 1% formaldehyde. At least 5 × 10^3^ cells/sample were analyzed by flow cytometry (Gallios Flow Cytometer; Beckman Coulter). DCF-DA fluorescence intensity was measured in FL-1 with an excitation wavelength of 488 nm and an emission wavelength of 530 nm.

### 4.4. T-Cell Priming Assay

To determine whether 7KCh- and Lupeol-treated DCs could prime naïve T lymphocytes, negatively selected naïve allogeneic T cells were co-cultured with the treated DCs at a 20:1 ratio. To exclude potential direct effects of the treatments on T cells, treated and untreated DCs were thoroughly washed before initiating co-culture experiments. Activated T cells were then expanded for 10 days in a complete medium, with recombinant IL-2 (30 U/mL; Roche Molecular Biochemicals, Indianapolis, IN, USA) added on day 5 in a 6-well plate. The resulting polyclonal T cell lines were subsequently analyzed for cytokine expression (IL-4, IL-10, IL-17, or IFN-γ) by flow cytometry, following previously established protocols [51]. Briefly, cells were labeled with anti-human CD3 PerCP (clone UCHT1; 5 µL/10^4^ cells) for 30 min on ice. After surface staining, cells were fixed with FACS lysing solution and permeabilized using FACS permeabilizing solution (BD Pharmingen, San Diego, CA, USA). Intracellular staining was performed using optimally titrated monoclonal antibodies: anti-human IFN-γ APC (clone B27), anti-human IL-17A APC (clone BL168), anti-human IL-4 PE (clone MP4-25D2), and anti-human IL-10 PE (clone JES3-19F1), or the corresponding isotype controls. All antibodies were purchased from BioLegend (San Diego, CA, USA). Sequential gating was used to determine the frequency of single- and double-positive cytokine-producing CD3+ T cells (see Supplementary Figure S1).

### 4.5. Cytokine Secretion Analysis in Dendritic Cell Culture Supernatants

Immature DCs were seeded in 24-well flat-bottom tissue culture plates (Corning, Costar Tewksbury, MA, USA) at a concentration of 0.8 × 10^6^ cells/mL and subsequently stimulated as previously described for 18 h. The concentrations of IL-12p70, IL-10, TNF-α, and IL-1β in the DC culture supernatants were measured using ELISA (OptEIA kits; BD-Biosciences, San Jose, CA, USA), following the manufacturer’s instructions.

### 4.6. Evaluation of NRF2/NQO1/HO-1 Pathway

#### 4.6.1. Indirect Immunofluorescence Labeling

For immunofluorescence microscopy, cells were plated on chamber slides (Millicell EZ SLIDE 8-well glass from Millipore, Milan, Italy) at a density of 80 × 10^3^ cells/well and were fixed after 18 h of treatment with 4% paraformaldehyde (Sigma-Aldrich) at room temperature for 30 min. After washing with PBS, cells were permeabilized with 0.1% Triton-X100 (Sigma-Aldrich) in PBS for 5 min at room temperature. Cells were then washed with PBS containing 0.05% Tween 20 (TBS-T), blocked with 0.1 M glycine in PBS for 20 min, and incubated with the primary antibodies: rabbit polyclonal anti-HO1 (1:500; Bioss antibodies, Beijing, China) and mouse anti-NRF2 (1:200; clone A-10, Santa Cruz Biotechnology, Dallas, TX, USA) in PBS at 37 °C for 90 min. After washing with PBS-T, fluorescence-labeled secondary antibodies were applied and incubated at 37 °C for 30 min: Alexa Fluor 488 goat anti-rabbit (Thermo Fisher Scientific, Milan, Italy) or Alexa Fluor 594 goat anti-mouse at a 1:100 dilution (Invitrogen, Carlsbad, CA, USA). Finally, the cells were marked with DAPI (Thermo Fisher Scientific) to highlight the nucleus, and the glasses were mounted with aqueous mounting medium. Images were captured using an upright microscope (Nikon Eclipse Ni-U, 60× g magnification, Nikon Corporation, Minato, Tokyo, Japan). Nikon NIS-Elements 5.0 imaging software (Nikon Corporation) was used for collecting images and for post-acquisition processing. The analysis was carried out in triplicate from three different fields.

#### 4.6.2. Western Blot Analysis of Dendritic Cell Lysates

Cells were removed rapidly and homogenized on ice with lysis buffer (CelLytic buffer, Sigma-Aldrich) plus protease and phosphatase inhibitors (protease inhibitor cocktail: 1 mM sodium fluoride, 1 mM sodium orthovanadate, and 1 mM sodium molybdate; 1 mM phenylmethylsulfonyl fluoride; and 1 mM phosphoinositidase C; all chemicals from Sigma-Aldrich). Protein extracts were cleared by centrifugation, and 30 μg of protein was separated by electrophoresis in 4–15% Mini-PROTEAN^®^ TGX Stain-Free™ Precast Gel (Bio-Rad, Hercules, CA, USA). Immediately after electrophoresis, the gel was placed into a ChemiDoc MP imaging System (Bio-Rad Laboratories) and UV-activated based on the appropriate settings with Image Lab Software (Bio-Rad) to collect the total protein load image. The proteins were transferred via the TransBlot Turbo semi-dry blotting apparatus (Bio-Rad) onto PVDF membranes (Bio-Rad). The membranes were blocked with 5% (wt/vol) low-fat milk (Sigma-Aldrich) in Tris-buffered saline (137 mM NaCl, 20 mM Tris·HCl, pH 7.6) containing 0.05% Tween 20 (TBS-T) for 1 h at room temperature. The membranes were incubated overnight at 4 °C with the primary antibodies mouse anti-NRF2 (1:500; SC-365949, Santa Cruz Biotechnology), anti-KEAP1 (1:500; PA5-34454, Invitrogen), anti-HO-1 (1:500; Bioss antibodies), and anti-NQO1 (1:500, clone A180, Abcam, Waltham, MA, USA). Appropriate peroxidase-conjugated secondary antibodies (1:10,000; Bio-Rad) were used to detect the proteins of interest by enhanced chemiluminescence (WesternBright™ Sirius Highest sensitivity chemiluminescent HRP Substrate, Advansta, Menlo Park, CA, USA). As a loading control, we used housekeeping proteins, and membranes were reprobed with anti-β-tubulin or anti-β-actin monoclonal Abs (Sigma-Aldrich). Protein immunoreactive bands were recorded with the ChemiDoc Imaging System (Bio-Rad) and analyzed using the National Institutes of Health ImageJ 1.62 software by Mac OS X (Apple Computer International, Cupertino, CA, USA). The results were expressed as the ratio of target protein normalized to housekeeping protein control, allowing the assessment of the absolute value density of each band on the same gel.

### 4.7. Molecular Docking Studies

Molecular docking is a potent method for studying receptor-ligand interactions that complements wet lab findings. For molecular docking, the human KEAP1 Kelch domain structure (PDB ID: 1U6D) was sourced from the RCSB Protein Data Bank and subsequently optimized through energy minimization using the GROMOS96 utility (without reaction field) in Swiss-Pdb Viewer 4.0.1.48. The 3D compound structures of Lupeol (Compound CID: 259846) were then downloaded from PubChem. The structures of the ligands underwent a meticulous optimization process using the advanced molecular mechanics geometry optimization module in Avogadro 1.1.1, for energy minimization [52]. In this study, we utilized UCSF Chimera version 1.17.3 along with the AutoDock Vina module for an effective docking analysis, adhering to the default configurations [53]. To enhance our understanding of the docking results, we employed Discovery Studio 4.0 for visualization purposes [54]. We calculated the total intermolecular energy and estimated the free energy of binding, which provided valuable insights into the binding affinities. Each experiment demonstrated a root-mean-square deviation (RMSD) of ≤2 Å, and to ensure reliability, all experiments were conducted in triplicate. Using the MM/PBSA approach and the Poisson Boltzmann surface area equation from molecular mechanics, the binding free energy of the docked Lupeol was determined [55].

### 4.8. Statistical Analysis

The Shapiro–Wilk test was applied to test data normality. Normally distributed data were analyzed using an exploratory one-way ANOVA. If ANOVA showed a significant effect, a Tukey post hoc test or independent Student’s *t*-test was conducted for group comparisons. Values of *p* < 0.05 were considered statistically significant. All the statistical analyses were performed by GraphPad Prism 8.0 software (San Diego, CA, USA).

## 5. Conclusions

This study provides the first evidence that Lupeol directly modulates the phenotype and function of human DCs toward an anti-inflammatory and regulatory profile, even in the presence of the pro-inflammatory oxysterol 7KCh. Our results show that 7KCh promotes DC maturation and T inflammatory responses, reinforcing its pathogenic role in autoimmunity. In contrast, Lupeol activates the NRF2 pathway, enhances antioxidant defenses, reduces oxidative stress, and shifts DC function toward anti-inflammatory cytokine production and Th2/Treg polarization. These findings highlight Lupeol’s dual immunomodulatory and antioxidant actions, supporting its development as a natural therapeutic agent for chronic inflammatory and autoimmune diseases. The ability of Lupeol to reprogram DCs in situ offers a feasible, non-invasive strategy to restore immune tolerance, potentially bypassing the limitations of current ex vivo DC-based therapies. Further studies should investigate its direct interaction with KEAP1 and assess delivery platforms to facilitate clinical translation.

## Figures and Tables

**Table 1:** Binding free energy of the complexes of Kelch domain with Lupeol given by the MM/PBSA method.

Energy (kcal/mol)	Kelch Domain-Lupeol
ΔE_ELE_	−197.52 ± 11.48
ΔE_VDW_	−134.43 ± 9.44
ΔE_GAS_	−331.95 ± 14.62
ΔG_NPS_	−25.10 ± 5.30
ΔG_PS_	301.04 ± 13.53
ΔG_SOL_	275.94 ± 16.42
ΔG_binding_	−56.01 ± 8.04 ^1^

^1^ Results are shown as the mean ± SEM of five sets.

## Data Availability

The raw data supporting the conclusions of this article will be made available by the authors, without undue reservation.

## Supplementary Materials

The following supporting information can be downloaded at: https://www.mdpi.com/article/10.3390/ijms26157179/s1.

## Abbreviations

The following abbreviations are used in this manuscript:

ANOVA	Analysis of Variance
APC	Antigen-Presenting Cell
CD	Cluster of Differentiation
DC	Dendritic Cell
FACS	Fluorescence-Activated Cell Sorting
H_2_DCF-DA	2’,7’-Dichlorodihydrofluorescein Diacetate
HLA-DR	Human Leukocyte Antigen—DR isotype
HO-1	Heme Oxygenase-1
iDC	Immature Dendritic Cell
IFN-γ	Interferon Gamma
IL	Interleukin
KEAP1	Kelch-like ECH-associated Protein 1
LPS	Lipopolysaccharide
Lup	Lupeol
MFI	Mean Fluorescence Intensity
ML385	NRF2 Inhibitor (specific small molecule inhibitor of NRF2 activity)
mTOR	Mechanistic Target of Rapamycin
NRF2	Nuclear Factor Erythroid 2–Related Factor 2
NQO1	NAD(P)H Quinone Dehydrogenase 1
ROS	Reactive Oxygen Species
SD	Standard Deviation
Treg	Regulatory T Cell
Th1/Th2/Th17	T Helper Cell Subtypes 1, 2, and 17
TolDC	Tolerogenic Dendritic Cell
t-BHQ	Tertbutylhydroquinone
7KCh	7-Ketocholesterol
