# Supplementary material for: Lupeol Attenuates Oxysterol-Induced Dendritic Cell Activation Through NRF2-Mediated Antioxidant and Anti-Inflammatory Effects
Source: Int J Mol Sci. 2025 Jul 25;26(15):7179. doi: 10.3390/ijms26157179 (PMC12346829; doi:10.3390/ijms26157179)
Supplement: Supplementary file 1 [file ijms-26-07179-s001.zip › ijms-3681162-supplementary.pdf]

Supplementary Figures

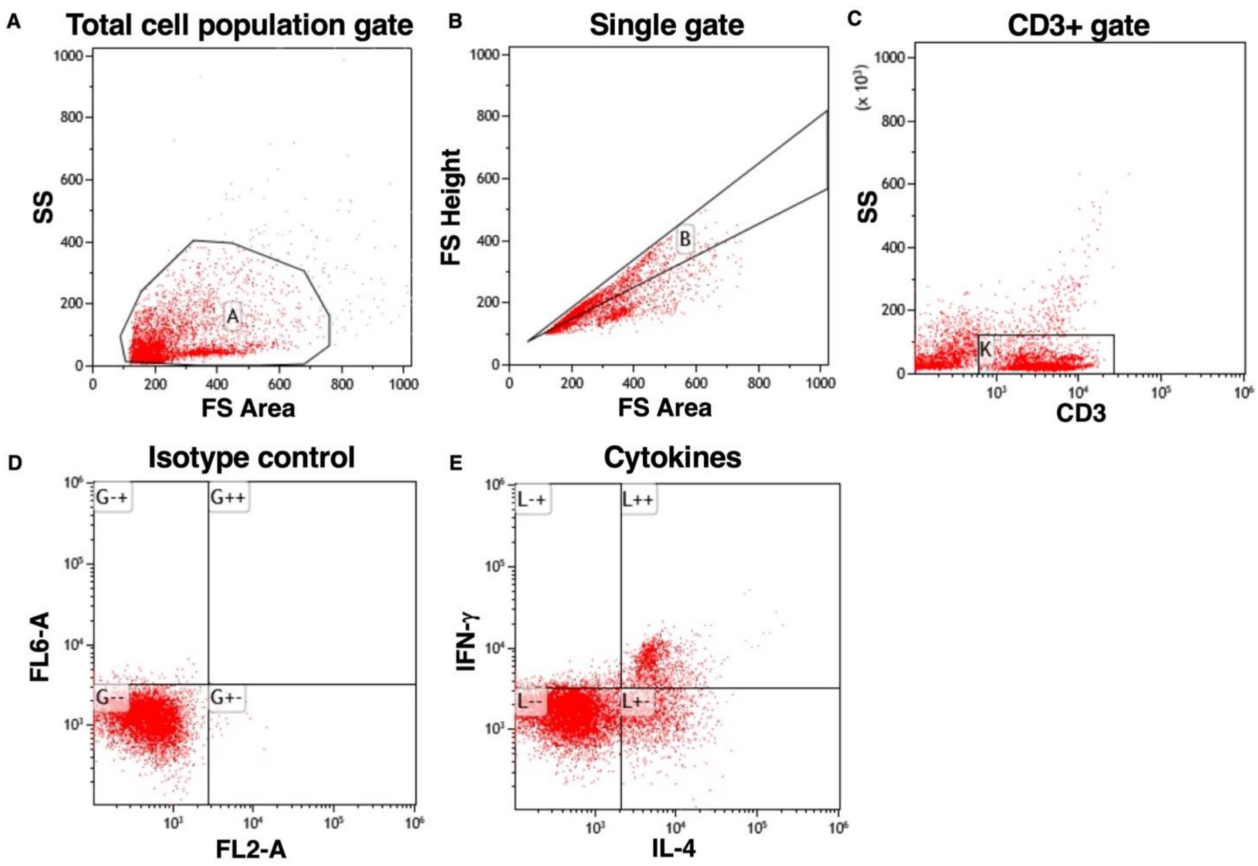

Legend for Supplementary Figure S1. Sequential gating strategy used for flow cytometric analysis of intracellular cytokine production in $CD3^+$ T cells. (A) Gate on total cell population based on size and granularity. (B) Singlet discrimination based on FSC-H vs. FSC-A. (C) Identification of $CD3^+$ T cells based on SSC vs. CD3 fluorescence. (D) Representative plot showing comparison with isotype control antibodies to define cytokine-positive populations. (E) Quantification of cytokine-producing subsets (e.g., IFN-γ^+, IL-4^+, single- and double-positive cells) within the $CD3^+$ gate.
